# Functional brain connectivity in young adults with post-stroke epilepsy

**DOI:** 10.1093/braincomms/fcad277

**Published:** 2023-10-18

**Authors:** Esther M Boot, Quinty P M Omes, Noortje Maaijwee, Pauline Schaapsmeerders, Renate M Arntz, Loes C A Rutten-Jacobs, Roy P C Kessels, Frank-Erik de Leeuw, Anil M Tuladhar

**Affiliations:** Donders Institute for Brain, Cognition and Behaviour, Department of Neurology, Radboud University Medical Centre, Nijmegen 6525GA, The Netherlands; Donders Institute for Brain, Cognition and Behaviour, Department of Neurology, Radboud University Medical Centre, Nijmegen 6525GA, The Netherlands; Department of Neurology and Neurorehabilitation, Luzerner Kantonsspital Neurocentre, Luzern 16, Switzerland; Department of Medical Psychology, Deventer Hospital, Deventer 7416 SE, The Netherlands; Department of Neurology, Medisch Spectrum Twente, Enschede 7500 KA, The Netherlands; Department of Neuroscience, F. Hoffmann—La Roche Ltd, Basel 4070, Switzerland; Donders Institute for Brain, Cognition and Behaviour, Department of Psychology, Radboud University, Nijmegen 6525 GD, The Netherlands; Department of Medical Psychology and Radboudumc Alzheimer Centre, Radboud University Medical Centre, Nijmegen 6525 GA, The Netherlands; Vincent van Gogh Institute for Psychiatry, Venray 5803 AC, The Netherlands; Donders Institute for Brain, Cognition and Behaviour, Department of Neurology, Radboud University Medical Centre, Nijmegen 6525GA, The Netherlands; Donders Institute for Brain, Cognition and Behaviour, Department of Neurology, Radboud University Medical Centre, Nijmegen 6525GA, The Netherlands

**Keywords:** young stroke, post-stroke epilepsy, resting-state functional MRI, network analysis

## Abstract

Approximately 1 in 10 young stroke patients (18–50 years) will develop post-stroke epilepsy, which is associated with cognitive impairment. While previous studies have shown altered brain connectivity in patients with epilepsy, little is however known about the changes in functional brain connectivity in young stroke patients with post-stroke epilepsy and their relationship with cognitive impairment. Therefore, we aimed to investigate whether young ischaemic stroke patients have altered functional networks and whether this alteration is related to cognitive impairment. We included 164 participants with a first-ever cerebral infarction at young age (18–50 years), along with 77 age- and sex-matched controls, from the Follow-Up of Transient Ischemic Attack and Stroke patients and Unelucidated Risk Factor Evaluation study. All participants underwent neuropsychological testing and resting-state functional MRI to generate functional connectivity networks. At follow-up (10.5 years after the index event), 23 participants developed post-stroke epilepsy. Graph theoretical analysis revealed functional network reorganization in participants with post-stroke epilepsy, in whom a weaker (i.e. network strength), less-integrated (i.e. global efficiency) and less-segregated (i.e. clustering coefficient and local efficiency) functional network was observed compared with the participants without post-stroke epilepsy group and the controls (*P* < 0.05). Regional analysis showed a trend towards decreased clustering coefficient, local efficiency and nodal efficiency in contralesional brain regions, including the caudal anterior cingulate cortex, posterior cingulate cortex, precuneus, superior frontal gyrus and insula in participants with post-stroke epilepsy compared with those without post-stroke epilepsy. Furthermore, participants with post-stroke epilepsy more often had impairment in the processing speed domain than the group without post-stroke epilepsy, in whom the network properties of the precuneus were positively associated with processing speed performance. Our findings suggest that post-stroke epilepsy is associated with functional reorganization of the brain network after stroke that is characterized by a weaker, less-integrated and less-segregated brain network in young ischaemic stroke patients compared with patients without post-stroke epilepsy. The contralesional brain regions, which are mostly considered as hub regions, might be particularly involved in the altered functional network and may contribute to cognitive impairment in post-stroke epilepsy patients. Overall, our findings provide additional evidence for a potential role of disrupted functional network as underlying pathophysiological mechanism for cognitive impairment in patients with post-stroke epilepsy.

## Teaser

Boot *et al*. report a less-efficient functional brain network in young patients with post-stroke epilepsy compared with those without. These alterations, particularly involving contralesional brain regions, may contribute to cognitive impairment, suggesting a disrupted functional network as a potential pathophysiological mechanism for cognitive impairment in post-stroke epilepsy.

## Graphical Abstract

**Graphical Abstract:**
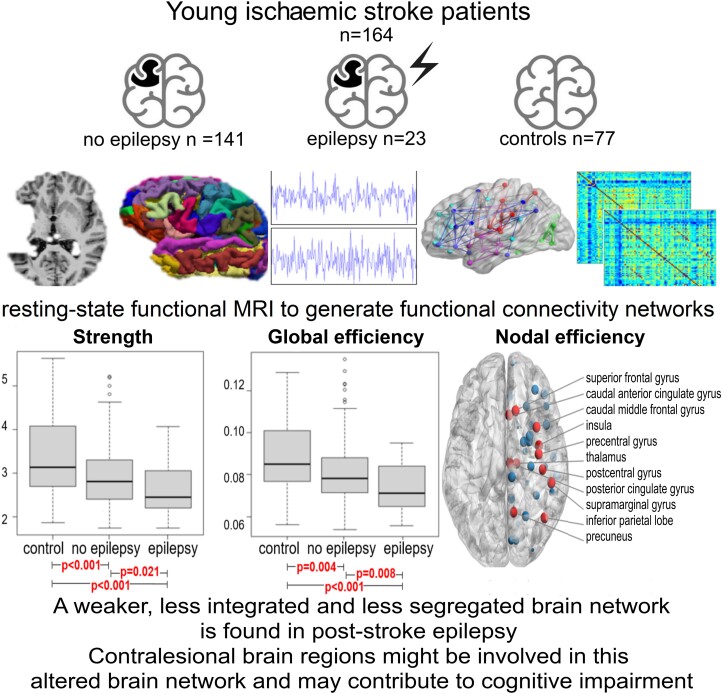

## Introduction

Stroke at a young age (18–50 years) poses a major personal and socioeconomic burden, as many young stroke patients experience a variety of post-stroke sequelae even decades after the event, which include post-stroke cognitive impairment and epilepsy (PSE).^1^ Previous studies have shown that PSE in young adults is more common than formerly thought with a long-term incidence of 10% in young ischaemic stroke patients.^2,3^ Young adults with PSE have a higher morbidity and mortality compared with young adults without PSE, and PSE has a negative effect on quality of life.^2^ In addition, patients with epilepsy in general display cognitive decline across a wide range of cognitive functions.^4-6^ The underlying mechanisms of PSE and its association with cognitive impairment are incompletely understood. Studies have suggested that PSE and post-stroke cognitive impairment may result from secondary brain changes, including reorganization of the brain networks.^4,7,8^ However, imaging evidence is currently lacking to link the functional brain network changes to PSE in young ischaemic stroke patients.

Over the last decades, the brain has been considered a complex network that can be analysed using graph theory as a mathematical paradigm.^9,10^ Functional network connectivity of the brain can be assessed with resting-state functional MRI (rs-fMRI), which measures brain activity by blood oxygen level-dependent (BOLD) signals of different brain regions during a resting condition (e.g. task independent). Several spatially distinct brain regions exhibit similar temporal properties of the BOLD fluctuations. These are considered to be functionally connected, mediated by the direct and indirect white-matter (WM) connections.^11^ Several studies have shown an association between less optimized network organization, based on functional connectivity and changes in hub regions, and various forms of epilepsy,^12-17^ albeit not in patients with PSE.

Studies regarding focal and idiopathic generalized epileptic patients also demonstrated changes in the hub regions.^18,19^ Hubs are connector nodes that connect separate modules within a network, and therefore play a key role in integrating information across a network.^20^ It is hypothesized that hubs are involved in epilepsy in two separate ways.^21^ Local pathological hubs close to the epileptogenic focus contribute to the spreading of epileptogenic activity.^22^ Physiological hubs, mostly in the default mode network (DMN), may be involved in the cognitive impairment found in epilepsy patients.^23^ However, our understanding of altered functional brain networks, especially of the hub regions, after stroke is limited, as most studies did not investigate PSE and post-stroke cognitive impairment simultaneously. Young stroke adults are particularly interesting since less comorbidity is at play and therefore less confounding factors for especially cognition, such as small vessel disease or Alzheimer pathologies.

The aim of this study was therefore to investigate the functional brain connectivity in young ischaemic stroke patients with PSE compared with stroke patients without PSE and healthy age- and sex-matched, and epilepsy-free controls and its relation with cognitive performance. We hypothesized that functional network organization is less optimized in stroke patients with PSE compared with stroke patients without PSE in young adults and is associated with impaired cognitive performance.

## Materials and methods

### Participants

**Figure 1:**
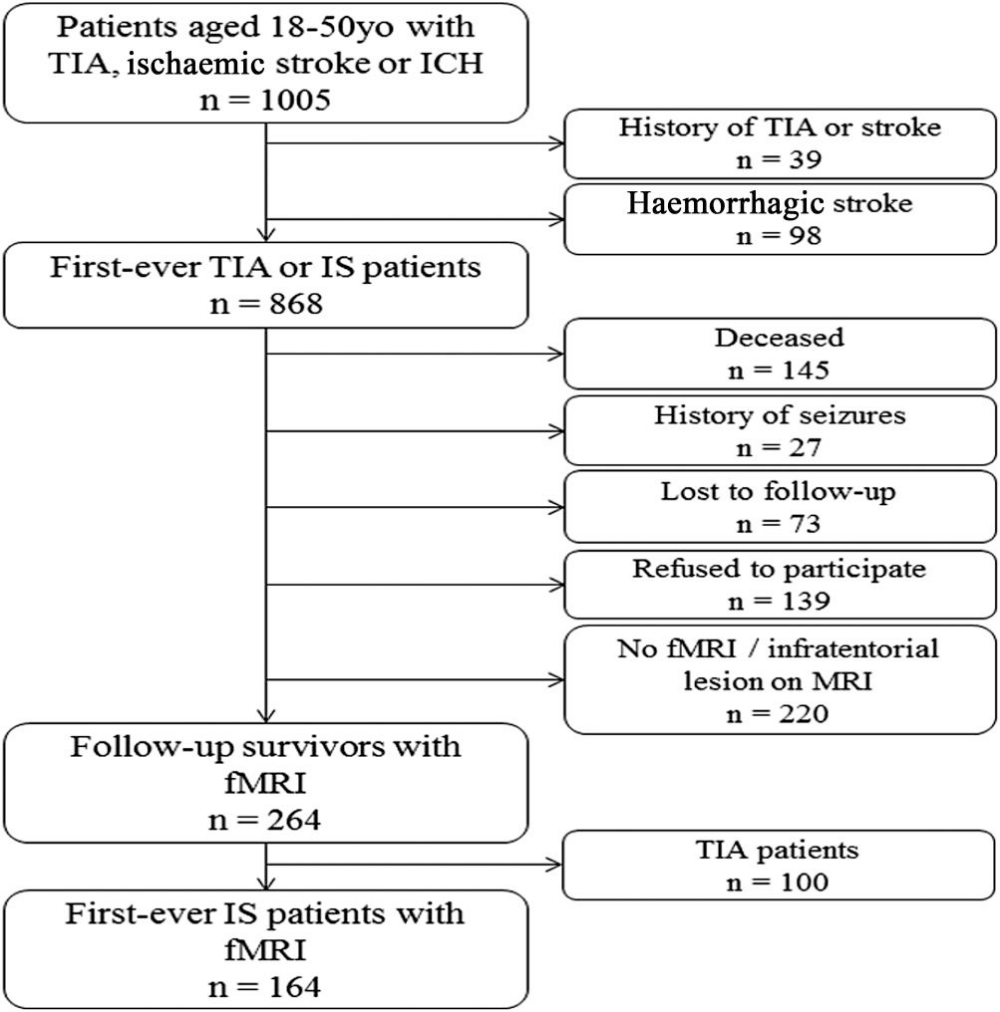
**Flow chart of the study population.** fMRI, functional magnetic resonance imaging; ICH, intracerebral haemorrhage; IS, ischaemic stroke; TIA, transient ischaemic attack.

This nested case–control study is a part of the Follow-Up of TIA and stroke patients and Unelucidated Risk factor Evaluation (FUTURE) Study, a prospective cohort study that investigates the causes and consequences of a stroke in young patients.^24^ Inclusion criteria were the presence of a transient ischaemic attack (TIA), ischaemic stroke of presumed arterial origin or intracerebral haemorrhage, that occurred between 1980 and 2010 in young adults aged 18–50 years and who visited Radboud University Nijmegen Medical Centre. During the follow-up between 2009 and 2012 participants underwent neuropsychological assessment and MRI scanning. For this study, we only included first-ever ischaemic stroke participants with an (f)MRI at follow-up. We excluded all participants with a history of epilepsy prior to the stroke. This resulted in 164 participants in the current study (Fig. 1). Controls were recruited among the participants’ spouses, relatives, or social environment. Inclusion criteria for controls were: age between 18 and 50 years old without a history of any TIA or stroke or epilepsy at the moment of inclusion. The controls were age and sex matched, without history of epilepsy. This resulted in 75 healthy controls.

### Standard protocol approvals, registrations and patient consents

All participants gave written informed consent according to the Declaration of Helsinki. The medical ethics committee region Arnhem–Nijmegen approved the study.

### Post-stroke epilepsy

We defined PSE according to the most recent definition of The International League Against Epilepsy.^25^ In this definition, epilepsy is diagnosed after a single seizure if there is an enduring condition with a higher risk of seizures (such as stroke).^26^ PSE is divided into acute and late onsets. Acute onset epilepsy is defined as seizures arising within 7 days after the stroke event. Consequently, late onset refers to seizures occurring later than 7 days post stroke.^27,28^ Furthermore, division was made based on a single seizure or recurrent seizures.

### Neuropsychological screening

Neuropsychological tests covered the main cognitive domains: processing speed (the written administration of the Symbol-Digit Modalities Test, Abbreviated Stroop Colour Word Test, Parts I and II), visuoconstruction (Rey–Osterrieth Complex Figure—copy trial), working memory (Paper and Pencil Memory Scanning Test), immediate memory (Rey–Osterrieth Complex Figure—immediate recall and the total number of words immediately recalled in the three-trial version of the Rey Auditory Verbal Learning Test), delayed memory (delayed recall on the Rey–Osterrieth Complex Figure and the Rey Auditory Verbal Learning Test), attention (Verbal Series Attention Test) and executive functioning (Verbal Fluency and Stroop Interference). Detailed information on the neuropsychological examination can be found elsewhere.^24^ The mean raw cognitive test scores [±standard deviation (SD)] for each test were calculated, and these raw test scores were converted to *Z*-scores, using the mean and SD of the controls. *Z*-scores of tests covering the same cognitive domain were averaged and used a domain score. Cognitive impairment was defined as >1.5 SD below age-adjusted mean of controls.^29^

### MRI data acquisition

MRI scanning was performed on a 1.5 T Magnetom scanner (Siemens, Erlangen, Germany). The scanning protocol included: (i) whole-brain 3D T_1_ magnetization-prepared rapid gradient-echo (MPRAGE) sequence [repetition time (TR)/echo time (TE)/inversion time (TI) 2730/2.95/1000 ms, flip angle 7°, voxel size 1.0 × 1.0 × 1.0 mm]; (ii) FLAIR pulse sequences (TR/TE/TI 12 220/85/2200 ms, voxel size 1.0 × 1.2 × 3.0 mm, slice gap 0.6 mm); (iii) resting-state imaging using a gradient-echo planar imaging (EPI) (TR/TE 1870/35 ms, voxel size 3.5 × 3.5 × 3.0 mm, slice gap 0.5 mm).

### Anatomical processing and brain regions

All participants’ T_1_-weighted (T_1_w) images were processed using FreeSurfer.^30^ FreeSurfer is an automated software suite for processing T_1_w images that include: skull stripping, registration to standard space Montreal Neurosciences Institute (MNI), segmentation, surface reconstruction and cortical parcellation.^31^ Brain nodes were defined using the Destrieux parcellation,^32^ combined with subcortical nodes from automated segmentation. This yielded 82 regions (41 per hemisphere, the cerebellum was excluded), which were used as network nodes for further analysis.

### Functional connectivity network construction

**Figure 2:**
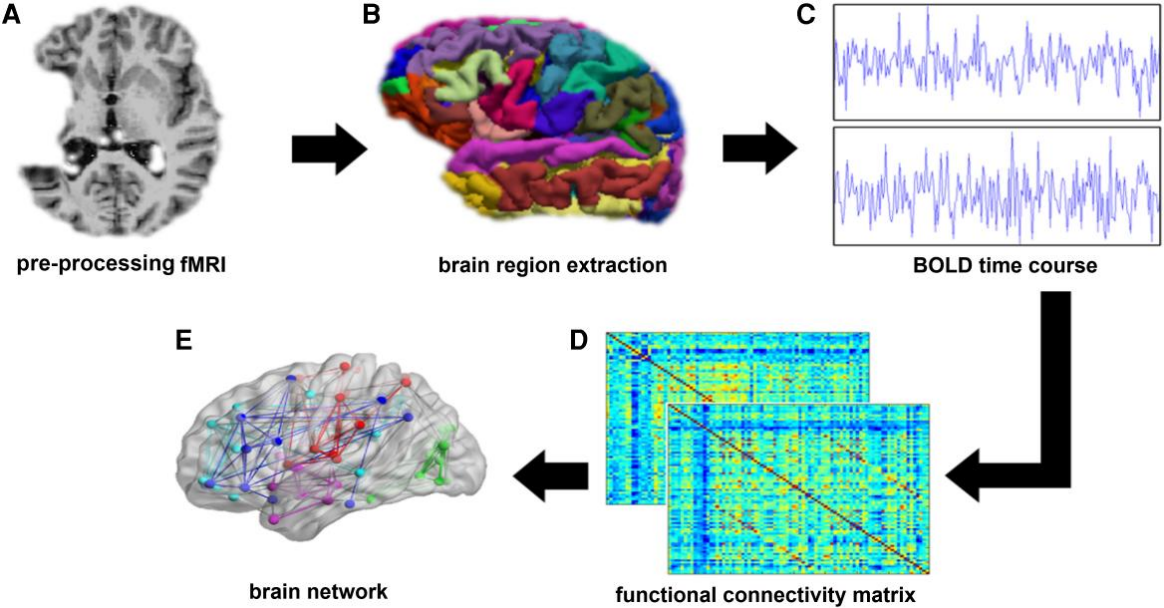
**The pipeline for processing resting-state fMRI (rs-fMRI).** (**A**) T_1_-weighted images and rs-fMRI were obtained from participants and controls. (**B**) After fMRI image pre-processing, 82 brain regions (41 in each hemisphere) were defined. (**C**) For each brain region, an average BOLD time course was extracted. (**D**) These BOLD time courses were used for the construction of connectivity matrices for each participants, and (**E**) brain network measures were calculated, on both global and regional levels. BOLD, blood oxygen level dependent; fMRI, functional magnetic resonance imaging.

The pipeline for processing rs-fMRI included (Fig. 2): (i) removal of the first four volumes of each acquisition to allow for steady-state magnetization, (ii) motion parameter estimation and realignment using Motion Correction using FMRIB’s Linear Image Registration Tool (MCFLIRT),^33^ (iii) brain tissue extraction using brain extraction tool, (iv) co-registration to the T_1_w image using boundary-based registration, (v) demeaning and linear detrending, (vi) temporal filtering using a Butterworth filter with a passband between 0.01 and 0.08 Hz and (vii) 36-parameter regression using 8 base regressors (the 6 rigid-body motion parameters estimated with MCFLIRT, mean WM signal, mean CSF signal), their first-order temporal derivatives, quadratic terms and the squares of their derivatives. To construct the functional connectivity matrix for each participant, the average BOLD time courses were extracted from each brain region. Pearson’s linear correlation coefficients were computed between the signals of every pair-wise region, which were then converted to *z*-values by applying a Fisher’s *r*-to-*z* transformation. Given the controversial nature of the physiological meaning of negative correlations,^34,35^ the elements of negative correlations were set to zero. The correlation matrices were then thresholded over the range of density values 0.05–0.40, with 0.05 increments. These matrices were then used to calculate integrated network measures from the area under the curve, resulting in less false-positive results coming from imperfect reconstruction of the connectome.^36^

### Graph theory analysis

Graph theoretical network measures were computed using the Brain Connectivity Toolbox for each participant’s weighted functional network.^37^ Assigning a weight to each edge results in additional information about the strength of connections and provides a more comprehensive understanding of network organizations.^38^

To characterize the global and regional functional network architecture, several topological properties of the functional brain network were calculated. This resulted in segregation properties (clustering coefficient, local efficiency and modularity), integration properties (global efficiency), centrality properties (betweenness centrality) and network strength for both the global whole-brain and regional functional network. The clustering coefficient measures the degree to which its neighbours tend to connect to each other. Local efficiency reflects the efficiency of the communication between two neighbouring nodes. These two parameters indicate the local ‘cliqueness’ of the network. Modularity quantifies the degree to which the network is partitioned into modules that have higher connections to each other than to the rest of the network. Betweenness centrality is based on the fraction of the shortest paths that pass through a certain node. Global efficiency was defined as the average inverse of the shortest path length. Nodal efficiency reflects the importance of a node in information exchange in the network. The strength of a node is the sum of the weights of the edges connected to that node.^37^

### Statistical analysis

Baseline characteristics were presented as mean ± SD for normally distributed data and median and interquartile ranges for the skewed parameters. We analysed group differences by using χ^2^-tests for categorical variables and two-sample independent *t*-test and one-way tests for the continuous variables, where appropriate.

We performed an analysis of covariance (ANCOVA), adjusted for age and sex, to examine between-group differences in global network measures for participants with and without PSE. For the global network measures that differed significantly between the group with PSE and the group without PSE, we additionally performed mass-univariate *t*-tests of brain region–specific network measures, adjusted for age and sex, to assess brain region differences between participants with and without PSE. To investigate the functional network changes in the brain, without the direct influence of the stroke lesion, the contralesional (e.g. ‘healthy’ or unaffected) hemisphere was used for these analyses. Analysing the contralesional hemisphere allowed us to make inferences about the remote influence of the stroke lesion, without distortion of the findings by the lesion itself. Hence, the participants with a bilateral lesion (*n* = 5) and participants without any lesion at follow-up MRI (*n* = 1) were excluded from the regional analysis. The significance level of *α* is set at 0.05. If a significant difference was determined in a certain brain region, we additionally performed general linear models using two models: in Model 1, we adjusted for age and sex, and in Model 2, we additionally adjusted for stroke lesion volume. To correct for multiple comparisons, a Benjamini–Hochberg false discovery rate (FDR) correction with a *q*-value of 0.05 was applied to the *P*-values.^39^

Next, we analysed group differences between participants with and without PSE for cognitive performance by using χ^2^-tests for impairment in cognitive domains and two-sample independent *t*-tests for the *Z*-scores of each cognitive domain. Furthermore, we used multivariate analysis for the significant cognitive domains, adjusted for age and stroke lesion volume. Finally, to test whether regional network measures could predict cognitive performance in PSE participants, we used linear regression models, with cognitive performance, using the *Z*-score of the significant cognitive domains, as dependent variable and global network measures of significant brain regions as predictor, adjusted for age, sex and anti-epileptic drug use.

All statistics were calculated using the statistical software R x64 4.0.0 and IBM SPSS Statistics 26.

## Results

### Group characteristics

In total, 241 participants were included in this study. Participants’ characteristics are summarized in Table 1. No significant differences in terms of age, sex or educational level were found between the three groups. Participants with PSE had larger stroke lesion volumes than participants without PSE (*P* = 0.00).

**Table 1:** Demographic and clinical characteristics

Clinical features	No epilepsy *n* = 141	Epilepsy *n* = 23	Controls *n* = 75
Mean age at follow-up, years (SD)	50.2 (9.1)	46.9 (9.6)	49.1 (11.6)
Gender, male *n* (%)	58 (41)	11 (48)	33 (44)
Educational level, low/high*^a^*	23/116	2/21	8/67
Mean follow-up, years (SD)	10.4 (8.0)	10.5 (8.1)	
IV rTPA treatment	4/137	2/23	
Lesion location, *n* (%)	*	*	
Left supratentorial	80 (0.57)	7 (0.30)	
Right supratentorial	57 (0.40)	14 (0.61)	
Bilateral supratentorial	3 (0.021)	2 (0.087)	
None	1 (0.007)	0 (0)	
Mean stroke lesion volume, ml (SD)	22.0 (40.7)**	71.4 (71.0)**	
TOAST			
Large artery	34 (24)	2 (9)	
Cardio-embolism	11 (8)	5 (22)	
Lacunar	24 (17)***	0 (0)***	
Other determined	23 (16)	4 (17)	
Multiple	3 (2)	1 (4)	
Underdetermined	46 (33)	11 (48)	
Onset epilepsy, *n* acute/*n* late		6/17	
Type of epilepsy			
Focal (%)		7 (30%)	
Focal impaired awareness (%)		2 (9%)	
Focal to bilateral tonic-clonic (%)		4 (17%)	
Generalized tonic-clonic (%)		9 (39%)	
Not otherwise specified (%)		1 (4%)	
Number of seizures			
1 (%)		15 (65%)	
2 (%)		8 (35%)	
Use of anti-epileptic drugs (Y/N)	5/136****	13/10****	
Number of anti-epileptic drugs*^b^*			
1 (%)		11 (86%)	
2 (%)		1 (8%)	
Cognitive impairment*^c^*	35/138	10/21	
Processing speed (%)	(25.4)*****	(47.6)*****	
Visuoconstruction (%)	26/139 (18.7)	8/23 (34.8)	
Working memory (%)	38/137 (27.7)	6/21 (28.6)	
Immediate memory (%)	26/139 (18.7)	4/23 (17.4)	
Delayed memory (%)	27/138 (19.6)	3/22 (13.6)	
Attention (%)	27/132 (20.6)	4/22 (18.2)	
Executive functioning (%)	26/138 (18.8)	6/22 (27.3)	

^a^Low education = 1–3 score, and high education = 4–7 score on a 7-point scale for education. ^b^Information about one patient is missing. ^c^Number of participants with impairment and number of participants without impairment. *χ^2^-test, *P* = 0.03. **Two-sample *t*-test, *P* = 0.003. ***χ^2^-test, *P* = 0.03. ****χ^2^-test, *P* < 0.00. ***** χ^2^-test, *P* = 0.04. IV rTPA, intravenous recombinant tissue plasminogen activator; TOAST, trial of ORG 10172 in acute stroke treatment.

### Global whole-brain analysis

**Figure 3:**
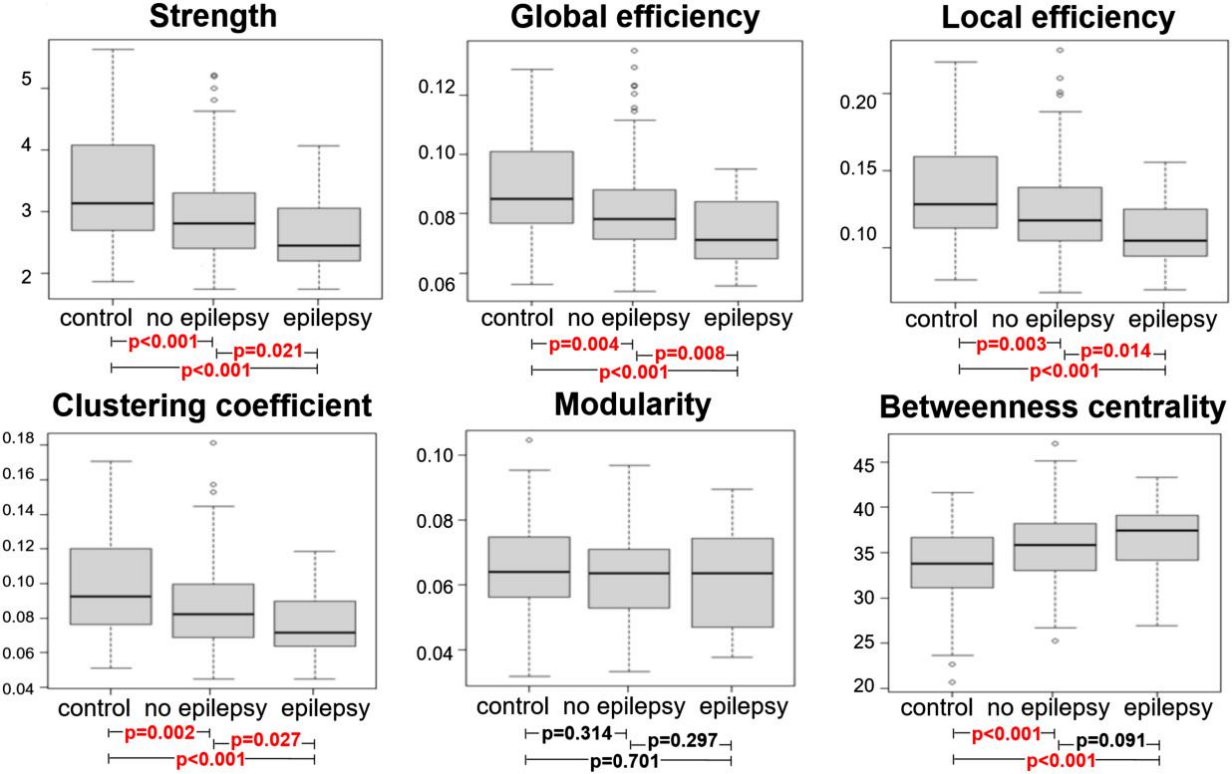
**Whole-brain network analysis.** Box plots of the functional network properties, divided by group (*n* = 241). Corresponding *P*-values computed with ANCOVA testing, adjusted for age and sex, are added below.

We found a decreased strength (*P* < 0.001), global efficiency (*P* = 0.001), local efficiency (*P* = 0.001), clustering coefficient (*P* = 0.001) and significant increased betweenness centrality (*P* < 0.001) in patients compared with controls. No difference in modularity was found between these groups (*P* = 0.30). Decreased strength of the functional network was found in participants with PSE compared with participants without PSE (*P* = 0.02). The PSE group exhibited a lower global efficiency (*P* = 0.01), lower local efficiency (*P* = 0.01) and decreased clustering coefficient (*P* = 0.03) compared with the participants without PSE. No differences in both modularity and centrality were found between these groups (*P* = 0.55 and *P* = 0.09, respectively). These results are plotted in Fig. 3. Furthermore, we found no differences in terms of global network measures between different aetiologies of stroke (*P* > 0.05).

### Acute versus late-onset epilepsy and single versus recurrent seizures

We performed a *post hoc* analysis in which participants with PSE with acute and late onset were compared, as well as participants with PSE with single and recurrent seizures. None of these demonstrated any between-group differences in the global network measures (*P* > 0.05). Furthermore, we performed linear regression models to examine whether time to the first seizure was associated with global network measures, adjusting for gender and age. This analysis showed that time to the first seizure was not a significant predictor for global network measures (*P* > 0.05).

### Regional analysis

**Figure 4:**
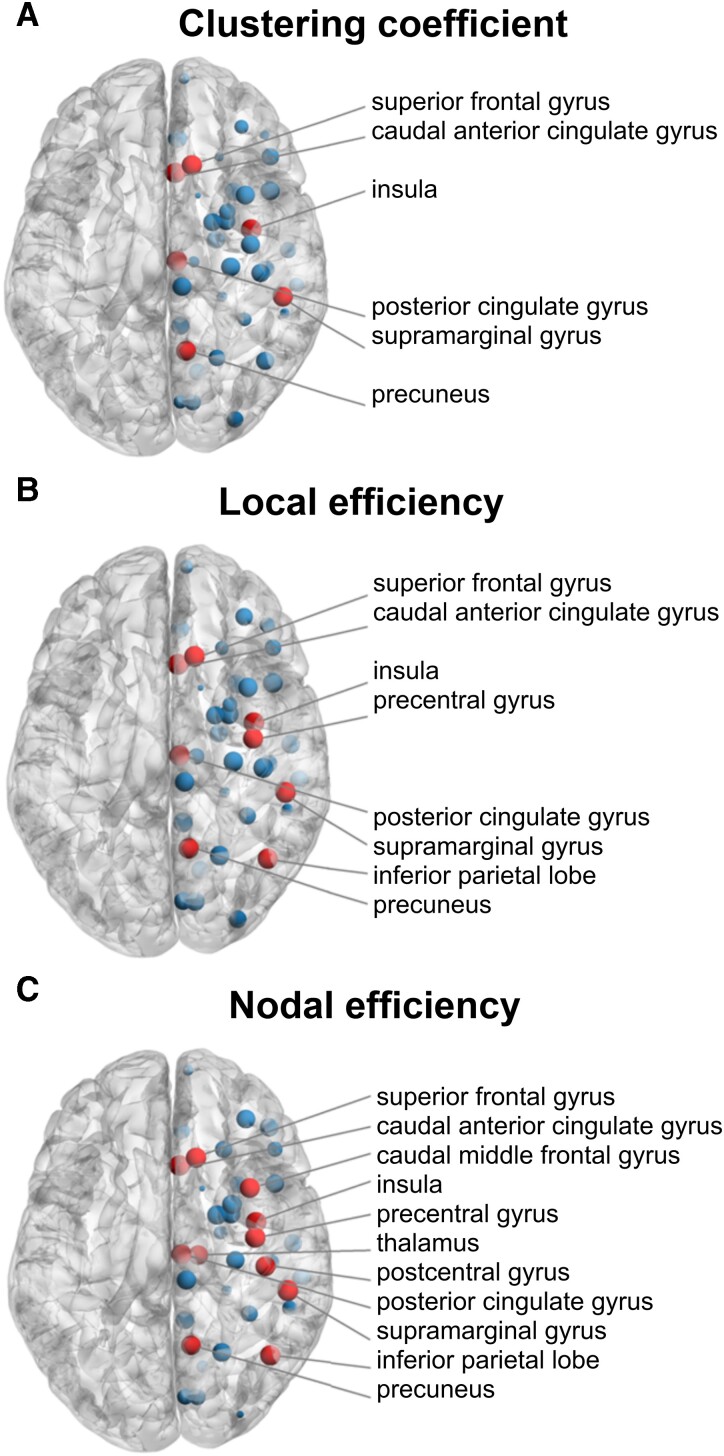
**Regional network analysis.** Brain regions (in red) of the contralateral (e.g. ‘healthy’) hemisphere, showing lower clustering coefficient (**A**), local efficiency (**B**) and nodal efficiency (**C**) in participants with PSE compared with participants without PSE (*P*-uncorrected < 0.05), for which general linear models were used (*n* = 158). After FDR correction for multiple comparisons, none of the brain regions remained significant.

Since global efficiency, local efficiency and clustering coefficient differed significantly between participants with and without PSE in whole-brain analysis, we examined nodal efficiency, local efficiency and the clustering coefficient at the regional level. For this analysis, we additionally excluded participants with a bilateral lesion (*n* = 5) and one participant without a lesion on follow-up MRI (*n* = 1), because analysis was performed on the contralesional (e.g. unaffected) hemisphere. This resulted in 158 participants included in this analysis, of whom 21 had PSE. Participants with PSE showed lower clustering coefficient in the caudal anterior cingulate cortex, the posterior cingulate cortex (PCC), the precuneus, the superior frontal gyrus and the insula compared with participants without PSE (all *P*-_uncorrected_ < 0.01; Fig. 4A). These brain regions also showed lower local efficiency (*P*-_uncorrected_ < 0.01), as did the inferior parietal lobe and supramarginal gyrus (Fig. 4B). A lower nodal efficiency (*P*-_uncorrected_ < 0.01) was found in the caudal anterior cingulate cortex, the PCC, the precuneus, the superior frontal gyrus, the insula, the inferior parietal lobe and supramarginal gyrus, the postcentral gyrus, the thalamus and the caudal middle frontal gyrus, in the PSE group compared with the group without PSE (Fig. 4C). These differences were significant after adjusting for age and sex (Table 2; Model 1) and in most regions, after adjusting for lesion volume (Table 2; Model 2), which did not survive FDR correction for multiple comparisons.

**Table 2:** General linear models for brain regions with significant differences between participants with and without PSE

Brain region	Model 1				Model 2			
	No PSE	PSE	*P*-value	FDR-corr. P-value	No PSE	PSE	*P*-value	FDR-corr. *P*-value
*Clustering coefficient*								
Caudal anterior cingulate cortex	0.08	0.06	0.01	0.10	0.08	0.06	0.02	0.33
Insula	0.08	0.06	0.01	0.10	0.08	0.06	0.03	0.43
Posterior cingulate cortex	0.09	0.07	0.01	0.10	0.09	0.07	0.01	0.33
Precuneus	0.10	0.09	0.03	0.25	0.10	0.09	0.05	0.43
Superior frontal gyrus	0.09	0.07	0.03	0.25	0.09	0.07	0.08	0.43
Supramarginal gyrus	0.09	0.07	0.05	0.32	0.09	0.07	0.07	0.43
*Local efficiency*								
Caudal anterior cingulate cortex	0.12	0.09	0.01	0.10	0.12	0.09	0.02	0.25
Inferior parietal lobe	0.15	0.13	0.04	0.21	0.15	0.13	0.08	0.32
Insula	0.12	0.09	0.00	0.10	0.12	0.09	0.03	0.25
Posterior cingulate cortex	0.13	0.10	0.02	0.10	0.13	0.10	0.02	0.25
Precentral gyrus	0.15	0.13	0.04	0.21	0.15	0.13	0.07	0.32
Precuneus	0.16	0.13	0.02	0.10	0.16	0.13	0.02	0.25
Superior frontal gyrus	0.15	0.12	0.01	0.10	0.15	0.12	0.03	0.25
Supramarginal gyrus	0.13	0.11	0.02	0.25	0.13	0.11	0.04	0.25
*Nodal efficiency*								
Caudal anterior cingulate cortex	0.08	0.06	0.00	0.062	0.08	0.06	0.01	0.17
Caudal middle frontal gyrus	0.09	0.08	0.04	0.164	0.09	0.08	0.05	0.17
Inferior parietal lobe	0.10	0.08	0.02	0.123	0.10	0.08	0.03	0.17
Insula	0.08	0.06	0.00	0.062	0.08	0.07	0.02	0.17
Postcentral gyrus	0.10	0.08	0.03	0.154	0.10	0.08	0.03	0.17
Posterior cingulate cortex	0.08	0.07	0.02	0.129	0.08	0.07	0.01	0.17
Precentral gyrus	0.10	0.08	0.01	0.11	0.10	0.08	0.01	0.17
Precuneus	0.11	0.09	0.04	0.164	0.11	0.09	0.04	0.17
Superior frontal gyrus	0.10	0.09	0.02	0.129	0.10	0.09	0.03	0.17
Supramarginal gyrus	0.09	0.07	0.01	0.082	0.09	0.07	0.03	0.17
Thalamus proper	0.06	0.05	0.04	0.164	0.06	0.05	0.06	0.17

FDR, false discovery rate; GLM, general linear model. Model 1: adjusted for age and sex. Model 2: adjusted for age, sex and stroke lesion volume.

### Regional network and cognitive performance in post-stroke epilepsy

Participants with PSE were more often impaired in the processing speed domain than participants without PSE (47.6 versus 25.4%, *P* = 0.04). Impairments in other cognitive domains did not differ significantly between the groups. Next, we performed linear regression models demonstrating that clustering coefficient [*β* = 0.47, 95% confidence interval (CI) 0.10–0.85, *P* = 0.02], local efficiency (*β* = 0.44, 95% CI 0.05–0.83, *P* = 0.03) and nodal efficiency (*β* = 0.45, 95% CI 0.09–0.82, *P* = 0.02) of the precuneus were significant predictors for the *Z*-score of processing speed in participants with PSE (*n* = 20, 1 participant without processing speed *Z*-score), adjusted for age, sex and use of anti-epileptic drugs.

## Discussion

In this study, we aimed to systematically assess the functional brain connectivity by applying graph theoretical framework on rs-fMRI in young ischaemic stroke patients with PSE, without PSE and healthy controls. Our main findings are that (i) global whole-brain analysis revealed a weaker, less-integrated (i.e. decreased global efficiency) and less-segregated (i.e. decreased clustering coefficient and local efficiency) functional network in participants with PSE compared with participants without PSE and healthy controls, (ii) in particular, altered functional connectivity of the brain regions in the contralesional hemisphere that belong to the DMN was observed in the participants with PSE and (iii) impairment of processing speed was more frequent in participants with PSE compared with participants without PSE, and at the regional level, the network properties of precuneus of the contralesional hemisphere were associated with processing speed.

The brain is considered to be efficient due to its small-world network organization.^20^ That is, a network with high efficiency and high clustering, which is essential as cognitive processes depend on a brain network with optimal organization of segregation and integration, rather than isolated brain regions. Our findings suggest that participants with PSE have a functional network that is less similar to a small-world network organization due to the lower integration and segregation compared with participants without PSE and healthy controls. This leads to a less-efficient functional network,^40^ which is consistent with findings of previous studies conducted in stroke patients.^41,42^ This may additionally explain why participants with PSE experience cognitive impairment, particularly in the domain of processing speed that is sensitive to whole-brain changes rather than localised lesions.

This finding is in contrast to studies with focal or idiopathic generalized epileptic patients that have demonstrated higher segregated network.^43-45^ It is hypothesized that the higher segregation in focal epilepsy patients might be the result of an adaptive process of the brain to inhibit the conversion of the interictal state to seizures, thus making it less susceptible to seizures.^45^ Our finding of an even lower segregation of the functional network in PSE patients compared with stroke patients without PSE may explain why these stroke patients are more susceptible to seizures. In contrast, the lower segregation could be the result of PSE due to maladaptive functional reorganization after stroke and the post-epilepsy adaptation towards a more segregated network has not yet taken place. This explanation is not likely as the stroke patients in our study were scanned more 10 years after the index event. Furthermore, previous research has shown that increased segregation was associated with diminished seizure frequency.^18^ However, we did not observe any significant differences between patients with recurrent epilepsy when compared with single seizure in our study, although it should be noted that the number of patients for this analysis is relatively low.

When comparing participants with PSE to participants without PSE by regional analyses, a decreasing trend of the clustering coefficient of the functional network was found in the caudal anterior cingulate cortex, the PCC, the precuneus, the superior frontal gyrus and the insula. These brain regions showed a decreased local efficiency of the functional network, as did the inferior parietal lobe and supramarginal gyrus. These brain regions are hub regions that especially belong to DMN.^46,47^ This is consistent with the finding that hubs are more likely to be abnormal than non-hubs in brain disorders.^48^ DMN is found to be less integrated in epilepsy patients interictally^49^ and was associated with intelligence as a measure of global cognitive function.^50^ Our findings imply that the precuneus, which is a part of the DMN, is involved in processing speed in PSE patients, which is in line with literature showing the relevant role of the DMN in information processing speed in healthy participants.^51^

Earlier research showed the influence of anti-epileptic drugs on functional network connectivity and on cognition. In our study population, the association between processing speed and the nodal efficiency of the precuneus, however, remained significant, after adjusting for AED, suggesting that the use of AED had little effect on the association between network measures and processing speed.^52-54^

In the regional analysis, the differences in these brain regions diminished after additionally adjusting for lesion volume. This decreased effect suggests that the stroke lesion at least partially determines the remote changes found in these brain areas located in the contralesional hemisphere. This is consistent with previous research in stroke patients that illustrated functional network changes in the contralesional hemisphere,^55^ implying that focal brain damage can have a widespread impact leading to altered brain reorganization, especially when hub regions are affected.^56^

Strengths of our study are the single-centre and prospective design, as well as the long follow-up period of 11 years, which enabled us to identify stroke patients with PSE. Furthermore, patients with first-ever ischaemic stroke were included in the study, which mitigates the risk that the epileptic seizure would be caused by pre-existing brain damage and not by the stroke index itself. In addition, patients with a history of epilepsy were excluded from the study.

This study has several limitations that should be addressed. First, the cross-sectional nature of this study prevents us from making any causal inference. The altered functional brain networks could be caused by stroke lesions, as stroke lesions were significantly associated with the functional network measures. Alternatively, PSE may induce functional reorganization leading to changes in functional connectivity. Second, the potential selection bias could be a possible limitation. In our study, we included all consecutive stroke patients at a young age. However, it could be that not all cases of young ischaemic stroke patients are referred to our hospital, as this is a single-centre study. As our hospital is the only university medical centre in our region, most young stroke patients are usually referred to our hospital during the course of the disease. Note that stroke patients were not referred to tertiary stroke centres for mechanical thrombectomy in the time period of the study. Third, several analyses were performed with a small subset of the study cohort and showed no significant differences. This might be due to the small sample sizes and therefore the lower power to detect any differences at the regional level, corrected for multiple comparisons. Despite these limitations, our results clearly showed that changes in functional connectivity are associated with PSE and cognitive deficits. Fourth, the node-specific results did not reach statistical significance after adjusting for multiple comparisons using FDR, and therefore, our conclusions are based on uncorrected results. While these results may risk false-positive errors, we believe they still contribute to our understanding of brain network alterations in the context of PSE, and our results can serve as hypothesis-generating findings for further research. Fifth, functional networks are presumed to be constrained by structural networks and may in turn exert an effect on structural connectivity via plasticity.^57^ Consequently, to make inferences about network alterations after stroke as a whole, data on functional and structural connectivity should be assessed simultaneously. Unfortunately, both data were only available for a very small subset of our cohort. Lastly, we used a technique based on BOLD fluctuations to assess the functional connectivity of patients with a neurovascular disease with potentially disrupted neurovascular coupling. To overcome fluctuations in blood oxygen levels due to disrupted neurovascular coupling, we investigated the contralesional hemisphere, which is unaffected by vascular lesions. Thus, we do not expect that our outcomes have been influenced by fluctuations as a result of a weaker neurovascular coupling system. These findings should be regarded as hypothesis generating and directing further research. Independent studies with a larger sample size of the epilepsy group and the subgroups, including recurrent and single seizure, are needed to confirm our findings.

## Conclusion

In this study, we demonstrated a weaker, less-integrated and less-segregated functional network in the participants with PSE compared with the participants without PSE and healthy controls. In participants with PSE, the hub regions may be particularly involved in lower integration and segregation and might play a role in cognitive performance, notable processing speed. Further longitudinal studies with larger sample size are needed to confirm our findings and prove the causal relationship. Changes in functional network measures of stroke patients have the potential to identify patients at risk of PSE and cognitive impairment that can be beneficial in more personalized decision-making.

## Data Availability

The data sets generated during and/or analysed during the current study are available from the corresponding author on reasonable request.
